# Ophthoselfie: Detailed Self-imaging of Cornea and Anterior Segment by Smartphone

**DOI:** 10.4274/tjo.66743

**Published:** 2017-06-01

**Authors:** Abdullah Kaya

**Affiliations:** 1 Dışkapı Yıldırım Beyazıt Training and Research Hospital, Ophthalmology Clinic, Ankara, Turkey

**Keywords:** Anterior segment, cell phone, imaging, selfie, ophthoselfie

## Abstract

**Objectives:**

To describe the ophthoselfie, a method by which everyone can take detailed self-images of the cornea and anterior segment with a smartphone.

**Materials and Methods:**

A 90-diopter non-contact double aspheric lens was attached to posterior camera of the smartphone by clear tape. Images of one eye on the screen of the smartphone could be seen with the other eye in the mirror and images were taken.

**Results:**

Accurate and detailed images of the cornea and anterior segment of the eye could be taken.

**Conclusion:**

The ophthoselfie allows everyone to take their own detailed anterior segment images by smartphone. To create a clear and detailed self-image of the cornea and anterior segment on the screen of a smartphone may lead to the development of new applications and facilitate patients’ early recognition of certain conditions like keratoconus, refractive errors, corneal rejection, and uveitis. This method may also be useful in some urgent situations by allowing patients to take self-images of the eye and share them with a physician.

## INTRODUCTION

It would be amazing if everyone could take their own detailed anterior segment images of the eye with a smartphone and share them with ophthalmologists or friends. Now it is possible with the ophthoselfie. With technological advancement, smartphones and social media have become essential components of our daily life. Smartphones provide possibilities to access the internet, take photos and videos, and share documents immediately via social media. Technological advancements have always had a major impact on medicine.^1^ Recording photos or videos of some pathologies is very important for medical education or follow-up survey of pathologies. Almost all physicians have a smartphone now. Thus, smartphones provide an opportunity to record certain pathologies immediately. Imaging is especially important in ophthalmology. A majority of diagnoses can be made by biomicroscopy. Camera systems can be attached to biomicroscopes in order to acquire images. However, it was not possible to have such technologies everywhere. Mohammadpour et al.^2^ identified a method that enabled the acquisition of ocular images by smartphone, without the use of a slit-lamp. They used a 90 diopter lens and were able to take detailed images of the anterior segment. The authors discussed the potential usage of this method by patients. Here I describe a method that provides an opportunity to take detailed self-images of the ocular surface, cornea, anterior segment and eyelids.

A selfie is a self-portrait photograph typically taken by a cell-phone camera held in the hand. It has become very popular in recent years. The ophthoselfie may also be a new trend among friends to share photos of the inside of their eyes and among patients to show their eye pathologies to ophthalmologists from afar.

## MATERIALS AND METHODS

**Figure 1:**
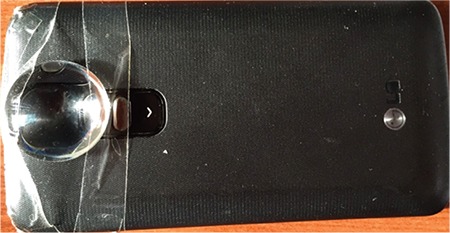
A 90 Diopter non-contact double aspheric lens was attached to the posterior camera of a smartphone using clear tape

**Figure 2:**
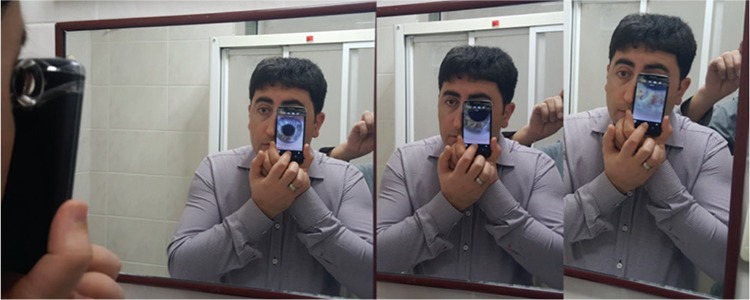
Taking an ophthoselfie facing a mirror. The screen of the smartphone can be seen in the mirror with the left eye and the most appropriate images are taken. Detailed anterior segment images including the iris crypts are visible

A smartphone with Android operating system (LG G2 mini, LG^®^ Electronics, South Korea) was used for imaging. A 90 diopter Volk^®^ (Volk Optical, Inc., Mentor, OH, USA) non-contact double aspheric lens was attached to the smartphone with clear tape (Figure 1). The focus setting was adjusted to manual, then the phone was passed in front of a mirror (Figure 2). As the camera was brought near one eye, the other eye could see the formation of the image in the mirror. The accuracy of the image could be arranged easily using the free eye.

## RESULTS

**Figure 3:**
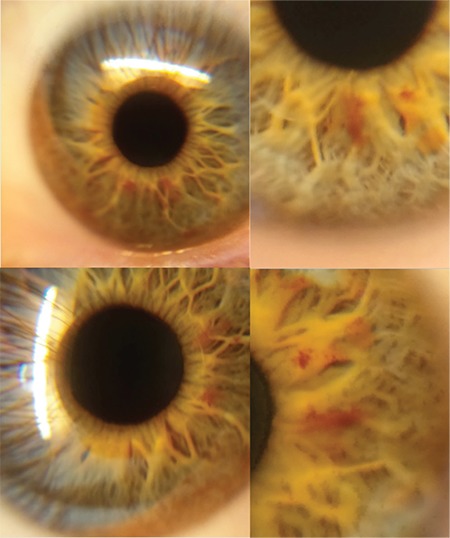
Ophthoselfies at different magnifications. Details of the cornea and anterior segment can be seen clearly. The images provide information similar to those taken with biomicroscopy

**Figure 4:**
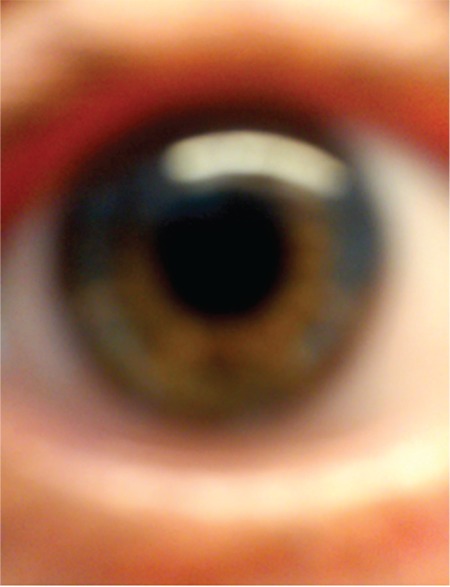
An image taken without using 90 diopter lens is not clear

Detailed self-images of the cornea and anterior segment could be taken by this method. High-resolution video was captured and even the iris crypts could be seen by adjusting the focus of the smartphone camera (Figure 3). An accurate image could not be taken without a 90 diopter lens (Figure 4).

## DISCUSSION

Self-imaging the eye was performed successfully using a smartphone and 90 Diopter lens while facing a mirror. This method allows even non-ophthalmologists to take images of the eye easily. Patients will be able to take images in cases of anterior segment traumas, blepharitis, hordeolum, keratitis, conjunctivitis, and hyphema. Furthermore, patients will be able to send postoperative images to their doctors. This will be a convenience to both patient and doctor. The ophthoselfie is not only an opportunity for patients but also for healthy individuals. While everyone can see their own eyes in the mirror, it is not possible to take a detailed image of the iris. The opportunity to take self-images of the inside of the eye and share them with friends may prove popular.

This method may be useful in some urgent situations by allowing patients to take self-images of the eye and share them with a physician. For example, corneal traumas or eye diseases like keratitis may happen to anyone while on vacation and an ophthalmologist is not readily accessible. Ophthoselfies may be life-saving in such a situation.

Although this method is user-friendly, it does have a limitation. The lens used in this study is a 90 diopter ophthalmic lens that is produced for ophthalmologists. These lenses are expensive and are not available everywhere. Special lenses that can be adapted to smartphones for this purpose may be developed.

## CONCLUSION

In conclusion, the main purpose of this study was to demonstrate the possibility of taking selfies of the eye. The ophthoselfie may become a popular phenomenon in the future.

## References

[1] Ozdalga E, Ozdalga A, Ahuja N (2012). The smartphone in medicine: a review of current and potential use among physicians and students. J Med Internet Res..

[2] Mohammadpour M, Mohammadpour L, Hassanzad M (2016). Smartphone Assisted Slit Lamp Free Anterior Segment Imaging: A novel technique in teleophthalmology. Cont Lens Anterior Eye..

